# Obesity is associated with quality of sperm parameters in men with infertility: a cross-sectional study

**DOI:** 10.1186/s12978-023-01664-2

**Published:** 2023-09-12

**Authors:** Mina Darand, Zahra Salimi, Moloud Ghorbani, Narges Sadeghi, Syavash Babaie, Mahdieh Hosseinzadeh

**Affiliations:** 1 Nutrition and Food Security Research Center and Department of Clinical Nutrition, School of Nutrition and Food Sciences, Isfahan University of Medical Sciences, Isfahan, Iran; 2 Nutrition and Metabolic Diseases Research Center, Ahvaz Jundishapur University of Medical Sciences, Ahvaz, Iran; 3 Department of Community Nutrition, Faculty of Nutrition and Food Sciences, Tabriz University of Medical Sciences, Tabriz, Iran; 4 Department of Nutrition, School of Public Health, Shahid Sadoughi University of Medical Sciences, Yazd, Iran; 5 Nutrition and Food Security Research Center, Shahid Sadoughi University of Medical Sciences, Yazd, Iran

**Keywords:** Obesity, BMI, WC, Sperm parameters, Male, Infertility

## Abstract

**Background:**

Previous studies examined the effects of obesity on sperm parameters and reported inconsistent results. Thus, the present study aimed to evaluate the association between obesity and the quality of sperm parameters in infertile men.

**Material and methods:**

The present cross-sectional study evaluated 218 infertile men aged 20–50. To this end, the 168-item food frequency questionnaire (FFQ) was utilized to evaluate dietary intake. The anthropometric and biochemical variables were examined using standard methods. Further, the association between obesity and the quality of sperm parameters was evaluated using the controlled linear regression for potential confounders.

**Results:**

The normal sperm morphology had a significant inverse association with BMI [adjusted β − 0.074, CI (− 0.141 to − 0.008), P = 0.029] and WC [adjusted β − 0.026, CI (− 0.051 to − 0.001), P = 0.038]. Additionally, visceral fat had a marginal inverse association with normal sperm morphology [adjusted β − 0.065, CI (− 0.138 to 0.008), P = 0.079] and non-progressive sperm motility [adjusted β − 0.241, CI (− 0.495 to 0.014), P = 0.063].

**Conclusion:**

Even though the present results indicated that obesity, abdominal obesity, and visceral fat had inverse associations with normal sperm morphology, more mechanism-based studies should be conducted to confirm these findings.

## Introduction

Obesity is a growing disease that is turning into a global epidemic worldwide [1]. It is defined as the accumulation of excess body fat, and a medical situation with negative effects on health and quality of life [2, 3]. According to the latest global reports, 1.9 billion adults are overweight and 650 million are obese [4]. The prevalence of overweight/obesity is 59.3% in Iran [5]. Obesity and its comorbidities, such as multiple metabolic and cardiovascular diseases, can increase mortality [6]. Obesity is also associated with other health outcomes such as infertility [7]. Based on the latest evaluations, infertility has involved approximately 48 million couples and 186 million individuals worldwide [8–10]. Male factor accounts for about 50% of overall cases of infertility. A recent meta-analysis of population-based studies reported that the prevalence of infertility was 7.88% in Iran [11]. Some of the well-known causes of male infertility include genital infections, testicular torsion or trauma, testicular varicocele, erectile dysfunction, hypogonadotropic hypogonadism, chronic and serious systemic disorders, obstruction of reproductive channels, and semen parameter abnormalities (motility, count, and morphology). Obesity is an important cause of male infertility [12–16]. Based on the evidence, overweight and obese men have lower sperm counts compared to their normal-weight counterparts [17–19]. Additionally, a high body mass index (BMI) has negative effects on sperm count, motility, morphology, and testosterone level [20, 21]. Some studies did not report any significant association between BMI and semen parameters [22–24]. It is postulated that obesity affects male fertility by reducing the testosterone level and the quality of semen [12]. Given that sperm motility has a greater association with the percentage of pregnancy and fertility rate than sperm concentration, motility abnormalities were the most common disorders in obese men with a prevalence of 39.9% and 24.2% in two studies [13]. Nevertheless, it is found that BMI optimization in obese men can improve sex hormone levels, erectile function, and semen parameters [25–28]. Given the few studies and their conflicting results, as well as the higher prevalence of infertility, the present study aimed to assess the association between obesity and sperm parameters quality in infertile men.

## Material and methods

### Study population

This cross-sectional study evaluated infertile men (aged 20–50 years) who received treatment at the Yazd Research and Clinical Center for Infertility, the main referral center for male infertility problems in southern Iran.

A total of 249 men were first included for participation in this study. The sample size was measured based on a 95% confidence interval and 80% test power and according to the correlation coefficient (r = 0.26) between WC and total sperm count in a study by Fejes et al. [29] according to the following equation:

N = [ (Zα + Zβ)/C]2 + 3.

The standard normal deviated for α = Zα = 1.9600. Further, the standard normal deviated for β = Zβ = 0.8416 and C = 0.5 * ln [ (1 + r)/ (1−r)] = 0.1820. Participants were selected using convenience sampling.

Exclusion criteria were no history of cryptorchidism, varicocele, microorchidism, vasectomy, or azoospermia, and disorders in morphology, motility, and concentration of sperm, such as chronic diseases, and genetic disorders. Additionally, participants who did not respond to more than 35 food items of the food frequency questionnaire (FFQ), or whose caloric intake was over 800–4200 kcal, were excluded from the study. A total of 218 eligible men were selected for the study after applying all inclusion and exclusion criteria.

### Ethics approval

The Ethics Committee of Shahid Sadoughi University of Medical Sciences approved the research protocol (approval code: IR.SSU.SPH.REC.1402.059). The present study was conducted based on the Declaration of Helsinki and all participants signed written informed consent forms before data collection.

### Anthropometric assessment

All anthropometric measurements were performed during the interviews using standard methods. Body height was also measured in a standing position using a tape measure on a straight wall to the nearest 0.5 cm. For height measurement, participants were barefoot and had their heads in the Frankfurt plane, shoulder blades, buttocks, and heels were contacted with the wall on which there was a tape measure. Body weight was measured using a Seca scale with an accuracy of 0.1 kg while participants stood in the center of the scale and unassisted with minimal clothing. Body mass index (BMI) (kg/m^2^) was also obtained from the weight and height measurements using the following equation.

$$\rm{BMI}=\rm{Weight} \, (\rm{kg})/\rm{Height} \, (\rm{m}^2)$$

Body composition (fat mass, muscle mass, visceral fat) was measured using a Tanita Body Scan (model 494, Tanita Corp., Tokyo, Japan). Waist circumference (cm) was obtained at an accuracy of 0.1 cm with a non-stretch tape measure without any pressure to the body surface midway between the last rib and the upper part of the pelvis at the end of a normal exhalation. When the measurement of the narrowest area of the participants' waist was difficult, the waist circumference was measured just below the last rib because the waist was likely to be the narrowest area between the iliac crests and the lower ribs in most participants [30].

### Physical activity, dietary assessment, and other covariate assessments

All participants completed questionnaires about demographic, medical history, education level, household income, and residential information. Moreover, physical activity levels were assessed using the International Physical Activity Questionnaire (IPAQ) [31] and converted to minutes per week (min/week).

Dietary intake was measured using a semi-quantitative food frequency questionnaire (FFQ) that was previously validated [32]. The FFQ comprised 168 food items that were filled out by a trained dietitian using face-to-face interviews. Participants were asked two types of questions about each food item: (1) The frequency of food consumption (number of consumption times daily, weekly, monthly, and annually) in the previous year, and (2) The amount of food that was consumed each time (based on standard Iranian serving sizes). All reported intakes were converted to g/day using household measures of consumed foods. Then, the average daily energy and nutrient intake were calculated using Nutritionist IV software that was modified for Iranian meals.

### Semen collection and analysis

Semen samples were taken by masturbation in the room near the laboratory and kept at 37 °C.

The participants were asked not to have ejaculation for at least 48 h before sampling. Semen analysis was performed based on the guidelines of WHO [33]. Sperm parameters such as sperm count (106/ml), motility, viability, and normal morphology were assessed for 200 spermatozoa for every sample. Sperm count and motility were also evaluated using the Makler chamber under light microscopy (Olympus Co., Tokyo, Japan). Further, the viability and morphology were assessed using the Eosin and Papanicolaou staining tests respectively.

### Statistical analysis

The participants' general characteristics were presented as mean (SD), and number (%) for quantitative and qualitative variables respectively. Linear regression was performed in crude and adjusted models to investigate the association between anthropometric parameters and body composition with sperm quality parameters. Adjustments were made for the confounding factors, namely energy intake, physical activity, age, and smoking. These confounding factors were extracted based on previous studies in this field. The independent t-test was also used to compare the quality of sperm parameters between obese and non-obese participants. P-value ≥ 0.05 was considered significant. All statistical analyses were performed using SPSS for Windows (SPSS ver. 20; SPSS Inc., Delaware).

## Results

Table 1 summarizes the participants' general characteristics. The mean age of 218 men, who participated in the study, was 33.77 years, and the mean of their BMI was 25.66 kg/m^2^. The mean waist circumference (WC), body fat percentage, muscle mass percentage, and visceral fat percentage were 93.71 ± 12.33 cm, 22.97 ± 8.15, 37.16 ± 4.69, and 8.44 ± 4.34%, respectively.

**Table 1:** Characteristics of the study participants

Variables	Total (n = 218) Mean ± SD or N (%)
Age (year)	33.77 ± 5.79
Energy intake (kcal)	3151.04 ± 630.62
BMI (kg/m^2^)	25.66 ± 4.79
WC (cm)	93.71 ± 12.33
Fat mass (%)	22.97 ± 8.15
Muscle mass (%)	37.16 ± 4.69
Visceral fat (%)	8.44 ± 4.34
Sperm concentration (million/ml)	36.08 ± 28.71
Sperm volume (ml)	3.40 ± 1.66
Sperm motility progressive (n)	30.82 ± 15.14
Sperm motility non-progressive (n)	10.77 ± 6.12
Sperm motility immotile (n)	58.58 ± 16.13
Sperm morphology (micrometer)	2.61 ± 1.67
Smoking, yes (%)	82 (37.6%)
Physical activity, N (%)	
Low	61 (28%)
Moderate	116 (57.4%)
High	25 (12.4%)
BMI categories	
Normal weight (BMI < 25)	93 (47.7)
Overweight/obese (BMI ≥ 25)	102 (52.3)

*BMI* Body Mass Index, *WC* waist circumference

Data are presented in quantitative variables as mean (SD) and for qualitative variables as number (%)

Table 2 presents the association between anthropometric parameters and body composition with sperm quality parameters. Based on this table, normal sperm morphology had a significant inverse association with BMI [adjusted β − 0.074, CI (− 0.141 to − 0.008), P = 0.029] and WC [adjusted β − 0.026, CI (− 0.051 to − 0.001), P = 0.038]. Further, visceral fat had marginal inverse associations with normal sperm morphology [adjusted β − 0.065, CI (− 0.138 to 0.008), P = 0.079] and non-progressive sperm motility [adjusted β − 0.241, CI (− 0.495 to 0.014), P = 0.063]. In other words, the higher percentage of visceral fat was associated with lower normal sperm morphology and slower non-progressive sperm motility. There was no significant association between semen parameters and other anthropometric measurements. Table 3 presents the mean and SD of sperm parameters using BMI and WC categories. The results of the intergroup analysis indicated significantly different percentages of normal sperm morphology among obese, non-obese men (P = 0.032), and those with and without abdominal obesity (P = 0.005). The percentage of normal morphology was lower in men with obesity or those with abdominal obesity.

**Table 2:** The association between quality of sperm parameters with energy intake, anthropometric parameters and body composition

Variables	Sperm concentration		Sperm motility progressive		Sperm motility non-progressive		Normal sperm morphology		Sperm volume	
	β (95% CI)	P-value^1^	β (95% CI)	P-value	β (95% CI)	P-value	β (95% CI)	P-value	β (95% CI)	P-value
*Energy intake*										
Crude	0.001 (− 0.005 to 0.007)	0.787	0.002 (− 0.001 to 0.006)	0.169	0 (− 0.001 to 0.002)	0.6	0 (− 0.001 to 0)	0.417	0 (0 to 0.001)	0.414
Adjusted model *	0.002 (− 0.004 to 0.008)	0.595	0.002 (− 0.001 to 0.005)	0.269	0 (− 001 to 0.002)	0.529	0 (− 0.001 to 0)	0.444	0 (0 to 0.001)	0.394
*BMI*										
Crude	0.197 (− 0.622 to 1.01)	0.636	0.491 (0.047 to 0.935)	0.030	− 0.156 (− 0.343 to 0.031)	0.101	− 0.038 (− 0.090 to 0.014)	0.149	− 0.001 (− 0.054 to 0.052)	0.982
Adjusted model ^†^	0.171 (− 0.877 to 1.23)	0.741	0.480 (− 0.096 to 1.05)	0.102	− 0.185 (− 0.421 to 0.051)	0.123	− 0.074 (− 0.141 to − 0.008)	**0.029**	0.010 (− 0.058 to.077)	0.781
*WC*										
Crude	0.076 (− 0.245 to 0.397)	0.641	0.132 (− 0.045 to 0.309)	0.143	− 0.048 (− 0.123 to 0.027)	0.209	− 0.015 (− 0.035 to 0.005)	0.133	0.005 (− 0.016 to 0.026)	0.645
Adjusted model^†^	0.091 (− 0.310 to 0.492)	0.653	0.087 (− 0.136 to 0.309)	0.443	− 0.051 (− 0.143 to 0.040)	0.271	− 0.026 (− 0.051 to − 0.001)	**0.038**	0.013 (− 0.013 to 0.039)	0.329
*Fat mass*										
Crude	0.359 (− 0.175 to 0.892)	0.186	0.181 (− 0.116 to 0.477)	0.231	− 0.057 (− 0.179 to 0.064)	0.352	− 0.001 (− 0.032 to 0.034)	0.966	− 0.027 (− 0.061 to 0.007)	0.118
Adjusted model^†^	0.367 (− 0.271 to 1.00)	0.258	0.154 (− 0.203 to 0.511)	0.395	− 0.097 (− 0.238 to 0.045)	0.179	− 0.010 (− 0.050 to 0.029)	0.610	− 0.024 (− 0.064 to 0.016)	0.245
*Muscle mass*										
Crude	− 0.667 (− 1.50 to 0.169)	0.117	− 0.059 (− 0.523 to 0.405)	0.802	0.076 (− 0.114 to 0.266)	0.431	0.005 (− 0.048 to 0.059)	0.852	0.015 (− 0.040 to 0.070)	0.595
Adjusted model^†^	− 0.603 (− 1.59 to 0.387)	0.231	− 0.012 (− 0.566 to 0.541)	0.965	0.094 (− 0.128 to 0.316)	0.405	0.022 (− 0.042 to 0.086)	0.502	− 0.002 (− 0.067 to 0.063)	0.948
*Visceral fat*										
Crude	− 0.667 (− 1.50 to 0.169)	0.117	− 0.059 (− 0.523 to 0.405)	0.802	− 0.076 (− 0.114 to 0.266)	0.431	0.005 (− 0.048 to 0.059)	0.852	0.015 (− 0.040 to 0.070)	0.595
Adjusted model^†^	− 0.378 (− 0.763 to 1.52)	0.514	0.522 (− 0.104 to 1.14)	0.102	− 0.241 (− 0.495 to 0.014)	**0.063**	− 0.065 (− 0.138 to 0.008)	**0.079**	− 0.024 (− 0.098 to 0.050)	0.522

^*^Adjusted for physical activity, age, and smoking

^†^Adjusted for energy intake, physical activity, age, and smoking

^1^Obtained from linear regression

**Table 3:** Quality of sperm parameters of men with and without obesity

Variable	BMI			WC		
	Non obese males (BMI < 30)	Obese males (BMI ≥ 30)	*P-*value*	Men without abdominal obesity (WC < 102)	Men with abdominal obesity (WC ≥ 102)	*P-*value*
Sperm concentration (million/ml)	36.37 ± 27.29	35.38 ± 27.78	0.851	35.11 ± 37.22	36.48 ± 27.19	0.760
Sperm volume (ml)	3.45 ± 1.77	3.32 ± 1.14	0.692	3.44 ± 1.79	3.51 ± 1.46	0.798
Sperm motility progressive (n)	29.66 ± 14.50	32.76 ± 17.22	0.282	29.52 ± 14.83	31.87 ± 15.57	0.343
Sperm motility non-progressive (n)	11.23 ± 6.62	9.48 ± 4.02	0.148	10.92 ± 6.39	11.10 ± 6.37	0.864
Normal sperm morphology (micrometer)	2.72 ± 1.80	2.19 ± 1.07	**0.032**	2.74 ± 1.77	2.10 ± 1.10	**0.005**

*BMI* Body Mass Index, *WC* waist circumference

^*^Obtained from independent t-test

## Discussion

The present study aimed to investigate the association between obesity and sperm quality in Iranian adults. Our results showed that higher BMI and WC were associated with lower normal sperm morphology, but sperm concentration, progressive motility, non-progressive motility, and volume had no association with obesity-related indices (BMI, WC, fat mass, muscle mass, and visceral fat). The finding also indicated that high visceral fat was to some extent associated with low non-progressive sperm motility and normal sperm morphology.

Obesity causes many health problems and has negative effects on fertility. Several review articles have examined the effects of obesity on the quality of sperm parameters and reported controversial results [22, 34–38]. Our findings were consistent with some studies in terms of some parameters [22, 34–36]. In a recent systematic review and meta-analysis, Salas-Huetos et al. reported that overweight and/or obesity were associated with low normal sperm morphology. Their result was consistent with our study. They also reported that overweight and/or obesity were negatively correlated with sperm count, concentration, and total motility [34]. In a meta-analysis of 15 studies with 6362 ordinary obese men, Wang et al. reported that obesity did not affect sperm concentration and percentage of normal sperm morphology, but significantly reduced total sperm number, and percentage of forward progression [35]. In a meta-analysis of 26,814 participants, Guo et al. reported that high BMI had no effect on sperm motility (overall or progressive), but significantly decreased sperm count and concentration [36]. In another meta-analysis by MacDonald et al., high BMI did not affect total sperm count or sperm concentration. Their result was consistent with our study [22]. Inconsistent with our results, a recent meta-analysis of 20,367 obese patients indicated that obesity was associated with lower sperm count, concentration, and progressive motility [38]. Further, Park et al. conducted a review article and reported that obesity was negatively correlated with sperm volume, concentration, motility, and count [37].

Several recent studies with interesting results have been conducted since the aforementioned meta-analyses [39–42]. For example, in a cross-sectional study, Esmaeili et al. found that BMI was negatively correlated with normal sperm morphology, sperm total motility, and progressive motility, but it had no effect on sperm volume and concentration. They also reported that waist circumference (WC), waist-to-hip ratio (WHR), skeletal muscle (SM), and visceral fat (VF) did not affect the quality of sperm parameters. Their result was consistent with our study [39]. Bahar GUR et al. also reported that higher VFT (visceral fat thickness) was negatively correlated with sperm normal morphology, but it did not affect sperm progressive motility and sperm concentration. They also found no significant correlation between BMI and sperm concentration, normal morphology, and progressive motility [40]. According to Abbasihormozi et al., high BMI was negatively correlated with sperm normal morphology, sperm motility, progressive motility, and sperm count [41]. Contrary to our results, Pooladi et al. reported a negative correlation between BMI and sperm motility (overall or progressive) but not sperm morphology and count [42]. Analyzing the studies, we found that the possible reasons for the inconsistencies might be related to different designs of studies, different sample sizes, different cut-off points of body mass index in determining obesity, and different health statuses of participants (infertile and healthy) in different studies. The possible mechanisms proposed for the relationship between obesity and the quality of sperm parameters are as follows.

One mechanism may be related to the disruption of the male reproductive endocrine axis that affects the regulatory function of the hypothalamic–pituitary–testicular axis. The low testosterone levels in obese men and the effect of testosterone on secondary spermatocyte meiosis and spermatocyte maturation may indicate the reduction of the semen volume and count [43, 44]. Additionally, the extra visceral adipose changes the hormonal milieu in males with obesity, thereby decreasing the SHBG level, free and total T, and inhibin B, and increasing T conversion into E2 because of higher aromatase activity [45]. Excessive visceral fat also creates insulin resistance and increases the insulin level. Consequently, the generation of SHBG in the liver decreases, leading to an increase in E2 level. The excessive E2 prevents the HPG axis and thus decreases the production of T [46]. Epigenetic modification also intensifies obesity via a variety of mechanisms, such as DNA methylation, modification of histone, and changes in miRNA, and can be transmitted to children [47, 48]. Moreover, the fat accumulation in the suprapubic region intensifies scrotal temperature, and increases in individuals with obesity, leading to impaired parameters in sperm and higher oxidative stress [49]. Pro-inflammatory cytokines are secreted by adipose tissue that causes low-grade systemic inflammation. Adipokine production also changes in obese individuals due to excessive leptin and leptin resistance. Therefore, male fertility decreases at peripheral and central levels [50]. In other words, Leptin modulates the production of GnRH through Kisspeptin and directly affects spermatogenesis [51]. Further, sirtuins mainly contributed to infertility caused by obesity. Sirtuin levels decrease significantly in fatty tissues in obese patients [48]. Sirtuins also control the testicular function by controlling diverse mechanisms that are essential for spermatogenesis [52]. The reduction of SIRT2 is associated with a decrease in GnRH, FSH, and LH generation with a change in spermatogenesis [53].

Unlike many studies on the association between obesity and sperm parameters using only BMI as an obesity index, the superior aspect of the present study was the investigation of the association of other obesity-related factors (WC, FM, Mm, VF) which might be more important than the body mass index in sperm parameters. Even though we could not achieve an effective association between some obesity-related factors and sperm parameters, we assume that the research hypothesis is defendable but needs further studies. Nevertheless, the present study had some limitations; first, the sample size was small. Second, it was impossible to estimate the causal relationship due to the cross-sectional design of the study. Further, more well-designed studies should be conducted to address male obesity and its effect on sperm parameters due to the lack of sufficient data on obesity-related parameters and sperm quality parameters.

## Conclusion

Even though the results of the present study indicated that BMI, WC, and visceral fat had inverse associations with normal sperm morphology, further mechanism-based studies should be conducted to confirm these findings.

## Data Availability

The datasets used and/or analysed during the current study are available from the corresponding author on reasonable request.

## Abbreviations

FFQ: Quantitative food-frequency questionnaire
MET: Metabolic equivalent
BMI: Body Mass Index
IPAQ: International Physical Activity Questionnaire
CI: Confidence interval
OR: Odds ratio
PUFA: Poly unsaturated fatty acids
DHA: Docosaexaenoic acid
EPA: Eicosapentaenoic acid
